# On-chip silicon photonic controllable 2 × 2 four-mode waveguide switch

**DOI:** 10.1038/s41598-020-80174-8

**Published:** 2021-01-13

**Authors:** Cao Dung Truong, Duy Nguyen Thi Hang, Hengky Chandrahalim, Minh Tuan Trinh

**Affiliations:** 1 Faculty of Electronics and Computer Engineering, Posts and Telecommunications Institute of Technology, Hanoi, Vietnam; 2 Department of Electrical and Computer Engineering, The US Air Force Institute of Technology, 2950 Hobson Way, Wright-Patterson Air Force Base, Dayton, OH 45433-7765 USA; 3 Department of Physics, University of South Florida, Tampa, FL 33620 USA

**Keywords:** Engineering, Optics and photonics

## Abstract

Multimode optical switch is a key component of mode division multiplexing in modern high-speed optical signal processing. In this paper, we introduce for the first time a novel 2 × 2 multimode switch design and demonstrate in the proof-of-concept. The device composes of four Y-multijunctions and 2 × 2 multimode interference coupler using silicon-on-insulator material with four controllable phase shifters. The shifters operate using thermo-optic effects utilizing Ti heaters enabling simultaneous switching of the optical signal between the output ports on four quasi-transverse electric modes with the electric power consumption is in order of 22.5 mW and the switching time is 5.4 µs. The multimode switch exhibits a low insertion loss and a low crosstalk below − 3 dB and − 19 dB, respectively, in 50 nm bandwidth in the third telecom window from 1525 to 1575 nm. With a compact footprint of 10 µm × 960 µm, this device exhibits a relatively large width tolerance of ± 20 nm and a height tolerance of ± 10 nm. Furthermore, the conceptual principle of the proposed multimode switch can be reconfigurable and scalable in multifunctional on-chip mode-division multiplexing optical interconnects.

## Introduction

Optical fiber communication systems and optical interconnects are now facing enormous demands on rapid bandwidth consumption of network traffics, especially in booming datacenters^1,2^. Besides, the growth of highly efficient computational systems leads to the increasing needs for high-bandwidth for information exchange centers^3^. To develop high-speed signal processing systems, some solutions have been proposed to scale the communication bandwidth aiming to overcome the barriers due to nonlinear limits^4,5^. Among them, the mode division multiplexing (MDM) technique has been considered as a promising solution for increasing communication bandwidth. In this method, the data was carried out using spatial orthogonality of guided modes. Since each mode is an independent channel thus potentially makes a myriad of single channel capacity for optical communications systems and optical interconnects when it combines with the wavelength division multiplexing (WDM) technique^6,7^. Various kinds of MDM systems have been proposed, for example, mode demultiplexer^8,9^, mode converter^10,11^, waveguide crossing^12,13^, mode selective switch^14,15^, mode add-drop multiplexer^16,17^, and mode router^18^.

In a multimode signal processing component, a multimode switch is one of the most important components to enable advanced processing functions for the MDM systems, especially, when combining MDM with WDM techniques, allowing an enhancement of data rates up to Tbps in a multi-mode waveguide that has been demonstrated in recent works^19,20^. The functionalities of optical mode switches have been achieved in on-chip photonic devices with both input and output signals in the single-mode mechanism^21,22^. In multimode optical communication, multimode optically switching is a big challenge in interconnect systems. The difficulty in making switching for multimode waveguides comes from the contradictory design requirements: that is the confinement of light in an optical multimode waveguide with different modal distributions causing the highly dimensional complexity of the waveguide structures for realizing switching functionalities. In a communication optical fiber, the low contrast of refractive indices between the core and the cladding layers ($\Delta n \approx 5.10^{ - 3}$) and the weakly guided modes make it difficult to separate the guided modes for processing individually. In contrast, for guided modes in an optical waveguide with a high refractive index contrast like silicon waveguides, ($\Delta n \approx 2$), the guided modes are strong. Moreover, the effective refractive index of silicon waveguides is highly independent on the modes so the interaction between guided modes is highly effective. Therefore, the modal transformation is also more flexible and convenient. An integrated multimode silicon waveguide could allow accessing specific modes for the reconfigurable switching system^23,24^. Currently, silicon waveguides widely use in the MDM system because of their advantages of wideband, high confinement of optical field, compact size, low power consumption, and especially CMOS-compatible devices^25^.

Many approaches have been reported to construct silicon few-mode selective switches (silicon MSS) for the configuration of MDM networks since the fundamental mode and the higher-order modes can convert into single mode, therefore these fundamental modes can be switched using spatial switching mechanisms. Stern et al*.*^26^ have proposed a structure based on microrings to capable of multimode switching for the first time but that structure only supports two modes and selected two dedicated wavelengths. Recently, Zhang et al. have successively demonstrated a silicon 2 × 2 four-mode dual polarization optical switch^27^ and a silicon 1 × 2 four-mode dual polarization optical switch^28^. However, both of those structures need asymmetric adiabatic couplers for realizing the (de)multiplexing functionalities in a multimode bus waveguide and Mach–Zehnder interferometers (MZI) for switching operation leading a large footprint and relatively complicated mechanism. Some other proposals for multimode switches either based on multiplexers/demultiplexers and waveguide crossing structures following Benes topologies^29,30^ or use lots of relatively complicated microring resonators and waveguide crossing elements.

In this paper, we propose a novel compact 2 × 2 four-mode optical switch enabling the switching operation of four modes simultaneously, which is based on Y-junction couplers and 2 × 2 multimode interference couplers. The proposed device could be constructed on silicon-on-insulator material and the switching operation of the device is performed via controllable phase shifters thanks to thermo-optic effect. The design, optimization, and characterization of the device are investigated using three-dimensional numerical simulations^31^ based on commercial simulation tools from Rsoft’s photonic device package.

## Working principle and optimization

**Figure 1:**
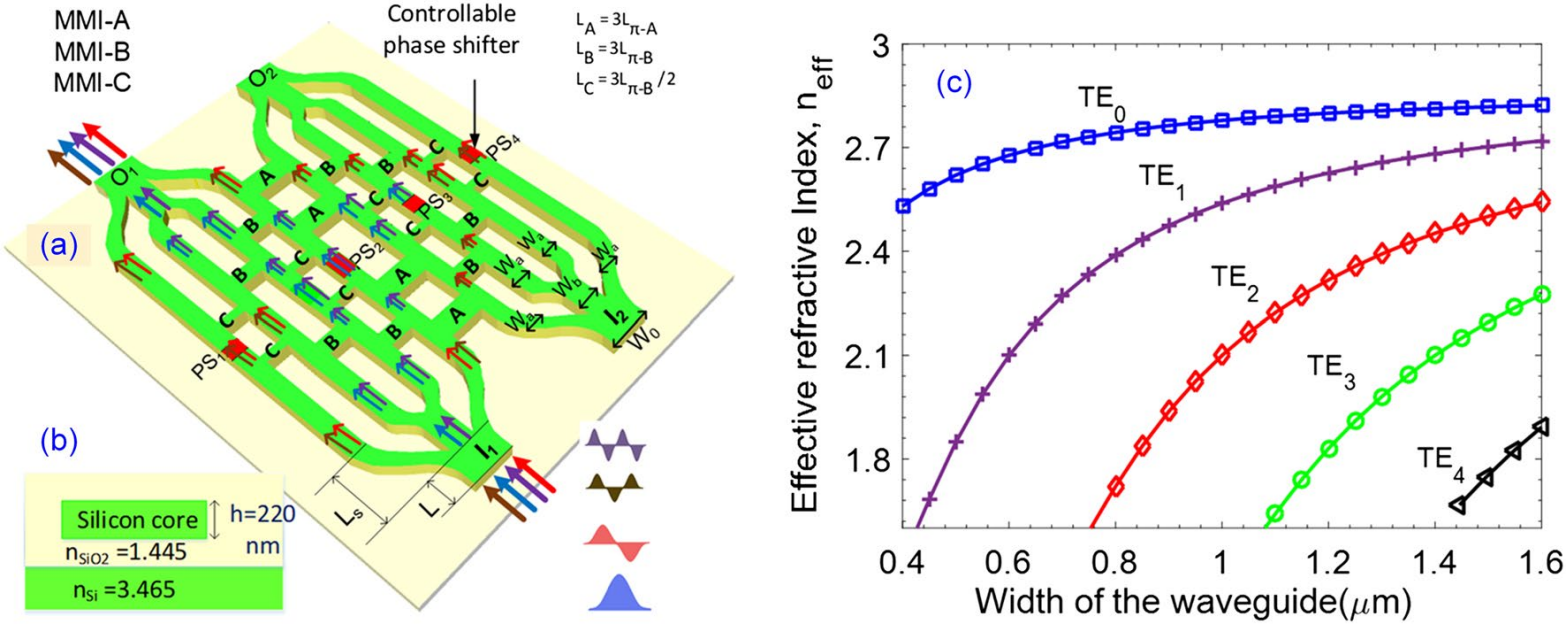
Device structure and TE mode characterization. Top view (**a**) and side view (**b**) of the conceptual diagram of the proposed four-mode switch. PS stands for phase-shifter. Three different couplers, MMI-A, -B, and -C, with the lengths denoted as L_A_, L_B_, and L_C_, respectively. (**c**) Effective indices for different guided modes of TE polarization as a function of input stem width via numerical simulation.

The working principle diagram of the proposed 2 × 2 multimode switch is shown in Fig. 1a,b. The proposed device consists of 4 identical and symmetric multi-branch 1 × 4 Y-junction couplers. The input sections have two 1 × 4 multi-branch Y-junctions that are symmetrically designed to guide the input multimode signals. The design uses three types of multi-mode interface (MMI) couplers composing of MMI-A, MMI-B, and MMI-C couplers. In this design, four MMI-A type couplers act as X-couplers between the two large symmetrical bridge of the device, eight MMI-B type couplers play the role of X-couplers between the internal branches, and eight MMI-C type couplers are 3-dB (50:50) couplers that divide and combine optical paths for the switching operations. Besides, the proposed device will enable us to simultaneously switch among four guided modes without blocking if we use four controllable phase shifters denoted PS_1_ to PS_4_ in order to combine suitable output optical signals.

The proposed device is constructed based on the structure of channel waveguides on silicon-on-insulator (SOI) material. The device is designed to support four transverse electric (TE) modes at the wavelength region of 1550 nm. The refractive index of the silicon core layer and the corresponding silicon glass cover are *n*_*r*_ = 3,465 and *n*_*c*_ = 1,445 at the wavelength of 1550 nm assume that these values do not change in the range of C-band.

**Figure 2:**
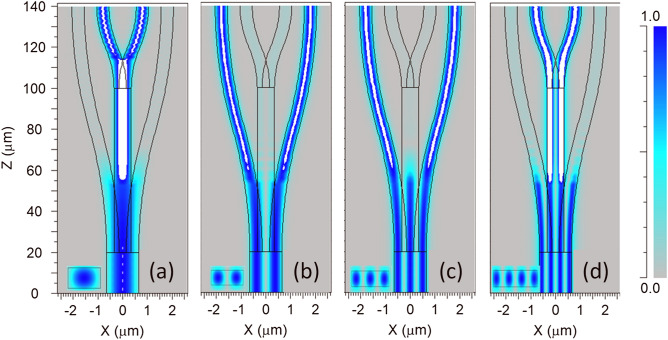
Electric field distribution. Numerical simulation of electric field distribution and mode solving calculation for the mode sorting in Y-multijunction waveguide for (**a**) TE_0_, (**b**) TE_1_, (**c**) TE_2_, and (**d**) TE_3_.

The 1 × 4 Y-junction coupler in this design composes of four sub-waveguides (as seen in Fig. 1a), in which, the main bus waveguide at the input, four S-bent waveguides, and one straight waveguide connecting two sections of Y-junctions. We use a numerical simulation tool of mode solver to model the effective indices of the four lowest order quasi TE modes from the input (including TE_0_, TE_1_, TE_2,_ and TE_3_ modes) as a function of waveguide stem width *W*_0_ of 1 × 4 Y-junction in the range from 0.3 µm to 1.6 µm, as presented in Fig. 1c. The simulated results show that the stem waveguide only guides enough four quasi-TE modes in the range from 1.1 to 1.45 µm. In this design, we have initially chosen the width *W*_0_ = 1.4 µm and the length of stem section *L* = 20 µm. The two S-bent waveguides on the two sides have the length in the propagation direction *L*_s_ = 120 µm and the width of two outer arms *W*_a_ = 0.5 µm. In the middle section, we use a straight waveguide whose width is *W*_b_ = 0.7 µm for supporting no more than two guided modes, which was utilized to connect to another Y-junction section with the width of outer arms as *W*_a_. These parameters were optimized using a mode-sorting technique, similar to the phase-matching method suggested by D. Love et al.^32^. Using these parameters, the stem of input waveguides would couple to the first and the second-order modes on two outer wings converting to the fundamental modes and then couple to the fundamental and the third-order modes in the middle straight branch which then continue to be divided to the fundamental modes at the outer wings of the second Y-junction substructure (O_1_ in Fig. 1a). Figure 2 presents the calculated mode field distributions at the input waveguide as well as the simulated electromagnetic field patterns of four guided modes when propagating through the 1 × 4 Y-multijunction that were optimized by numerical simulation corresponding to the fundamental and three remaining high order modes. In a 1 × 4 Y-junction, the fundamental mode and the third-order mode are distributed to two inner branches while the first and the second-order modes are distributed to two outer branches at the output.

Note that the even modes will be divided into the pairs of in-phase fundamental modes and the odd modes will be divided into the pairs of counter-phase fundamental modes. The optical modes then continue to be guided through the waveguides and recombined at the outputs of the 1 × 4 Y-multijunctions with appropriate phase shifts, which are driven via controllable phase shifters (PSs) for switching to the desired output ports of the switches.

**Figure 3:**
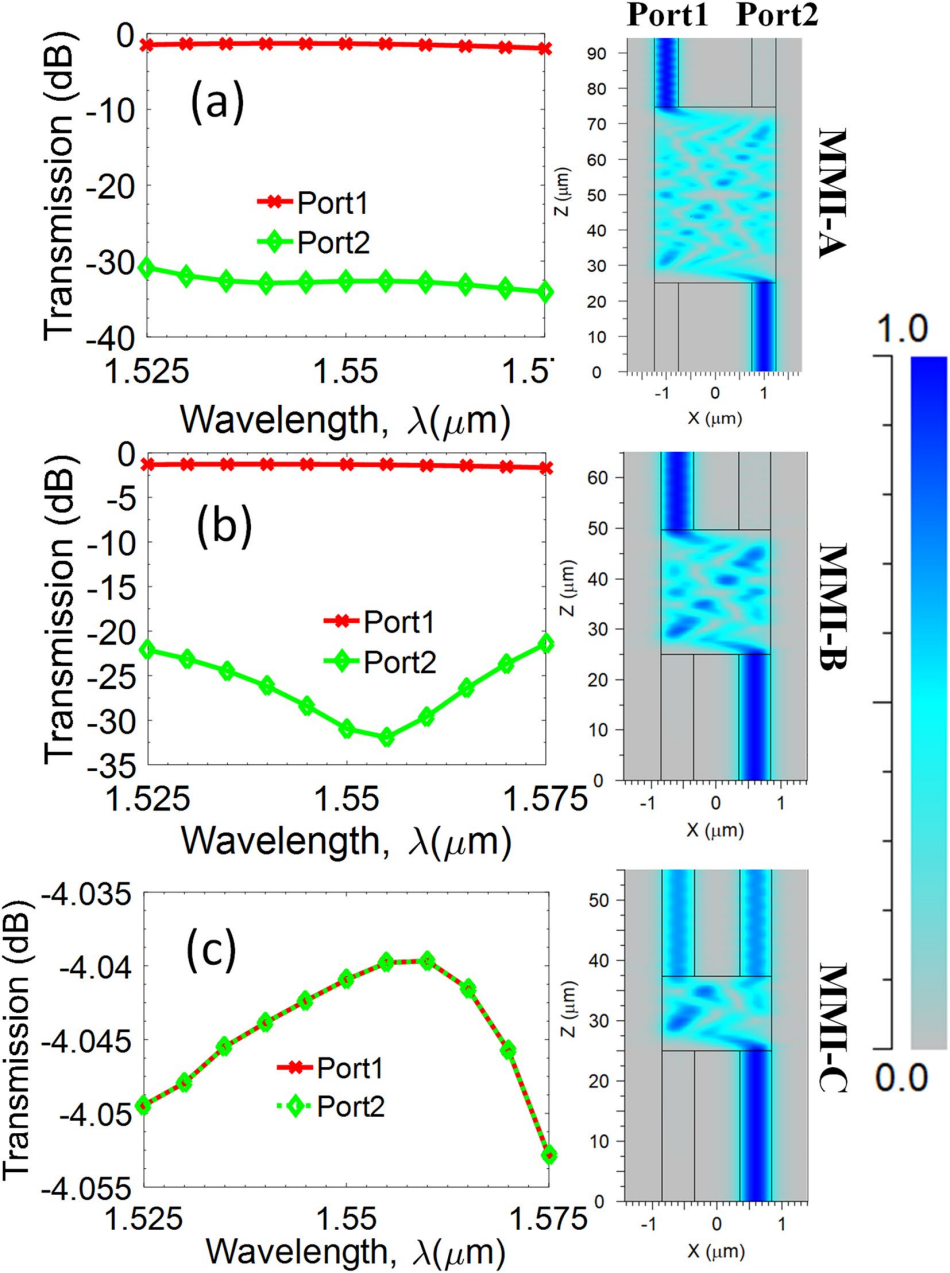
Transmission characteristics of three kinds of 2 × 2 MMI couplers on wavelength dependence. (**a**) MMI-A and (**b**) MMI-B are X-couplers with the lengths of $L_{MMI} = 3L_{\pi }$, (**c**) MMI-C is 3 dB-coupler with the length of $L_{MMI} = 3L_{\pi } /2$.

In this design, we used twenty 2 × 2 MMI couplers in three kinds named A, B, and C for switching operation (Figs. 1a, 3). Three kinds of multimode interference couplers have the widths corresponding to $W_{A}$, $W_{B}$, $W_{C}$ and the lengths corresponding to $L_{A}$, $L_{B}$, $L_{C}$, respectively. The working principle of a multimode interference coupler follows the Talbot effect^33^. In the general interference (GI) mechanism, the optical field is periodically reproduced along the propagation direction^34^. Self-imaging will be mirrored if the length of the multimode region $L_{MMI} = 3L_{\pi }$, in that case the MMI coupler will play the role of an X-coupler. Whereas, self-imaging will be a mirrored pair of a photograph if the multimode region length $L_{MMI} = 3L_{\pi } /2$, and the MMI coupler will work as a 3 dB-coupler. Where $L_{\pi }$ is the half-beat length which is defined as follows:

1 $$L_{\pi } = \frac{{4n_{{{\text{ef}}f}} W_{e}^{2} }}{3\lambda },$$

2 $$W_{e} = W_{MMI} + \frac{\lambda }{\pi }\left( {n_{eff}^{2} - n_{c}^{2} } \right)^{ - 0.5} ,$$

here, *W*_*e*_ is the effective width calculated by the osmotic depth of the transverse electric mode; $\lambda$ is the operation wavelength; $n_{eff}$ is the effective index; $n_{c}$ is the refractive index of the cladding layer.

In this design, MMI-A and MMI-B couplers are the X-couplers while the MMI-C is the 3 dB-coupler with the lengths that were optimized by numerical simulation. The transmission for wavelength responses in a band from 1525 to 1575 nm of three MMI couplers is presented in Fig. 3. For the X-couplers in Fig. 3a,b, the switching is very efficient with the output power at the desired ports of about − 1 dB and is wavelength independent while output powers at undesired ports are smaller than − 22 dB in the wide band of 50 nm. Figure 3c shows the transmission curves of the MMI-C coupler with the optical field is equally divided into two output ports. The transmission outputs from two ports are identical with a small variation of 0.01 dB in the range of 50 nm, therefore, this coupler is a perfect wideband 3-dB coupler.

In this section, we will discuss the use of controllable phase shifters to combine or separate the optical fields. In order to achieve desirable simultaneously switching operation, four controllable phase shifters need to place at four branches as seen in Fig. 1a. For each guided mode at the input port, after traveling through two different light paths in the circuit, will be recombined at the desired output port. Following the self-imaging principle when the multimode interference coupler in the role of X-coupler at the length of $L_{MMI} = 3L_{\pi }$, the image is a direct copy of the optical field at the input with the phase change of even or odd multiple of *π*. Hence, the interference mechanism occurring in this structure is similar to the interference mechanism in a Mach–Zehnder interferometer. Note that TE_0_ and TE_3_ modes are under influenced by the PS_1_ and the PS_4_, whereas, TE_1_ and TE_2_ modes are under influenced by the PS_2_ and the PS_3_. The dependence of the output powers on the phase shift angles of controllable PSs for four modes can be written by explicit functions as follows:

3 $$P_{pn} = \frac{{P_{in} }}{2}\eta_{c,p} 10^{ - \alpha L} \left( {\sin^{2} \left( {\frac{{\Phi_{2} }}{2}} \right) + \sin^{2} \left( {\frac{{\Phi_{3} }}{2}} \right)} \right),$$

4 $$P_{pn} = \frac{{P_{in} }}{2}\eta_{c,p} 10^{ - \alpha L} \left( {\cos^{2} \left( {\frac{{\Phi_{1} }}{2}} \right) + \cos^{2} \left( {\frac{{\Phi_{4} }}{2}} \right)} \right),$$

5 $$P_{qn} = \frac{{P_{in} }}{2}\eta_{c,q} 10^{ - \alpha L} \left( {\sin^{2} \left( {\frac{{\Phi_{1} }}{2}} \right) + \sin^{2} \left( {\frac{{\Phi_{4} }}{2}} \right)} \right),$$

6 $$P_{qn} = \frac{{P_{in} }}{2}\eta_{c,q} 10^{ - \alpha L} \left( {\cos^{2} \left( {\frac{{\Phi_{1} }}{2}} \right) + \cos^{2} \left( {\frac{{\Phi_{4} }}{2}} \right)} \right),$$

where $p = \left\{ {0,3} \right\}$ and $q = \left\{ {1,2} \right\}$ are the orders of guided modes; $n = \left\{ {1,2} \right\}$ stands for the orders of the output ports.$P_{in}$ is the input power of each mode; $\eta_{{c,\left\{ {p,q} \right\}}}$ are the coupling efficiencies of the *p,q-*th order modes when coupled with the waveguide. $\alpha$ is the loss coefficient of silicon at the operation wavelength $\lambda$, typically $\alpha = 1{\text{dB/cm}}$ for the wavelength of 1550 nm^35^. *L* is the total device length; $\Phi_{1,2,3,4}$ are the phase shift angles under the control of the corresponding phase shifters PS_1,2,3,4_, respectively.

In case all four controllable phase shifters are handled by only one external source such as a voltage-driven thermal source, the phase shift angles are the same: $\Phi_{1} = \Phi_{2} = \Phi_{3} = \Phi_{4} = \Phi$. Consequently, the combined optical fields at the output will be reformed to the mode shape like the input guided mode. Then, we can shorten the Eqs. (3,4,5,6) as:

7 $$P_{mn} \left( \Phi \right) = P_{in} \eta_{c,m} 10^{ - \alpha L} \sin^{2} \left( {\frac{\Phi }{2}} \right),$$

**Figure 4:**
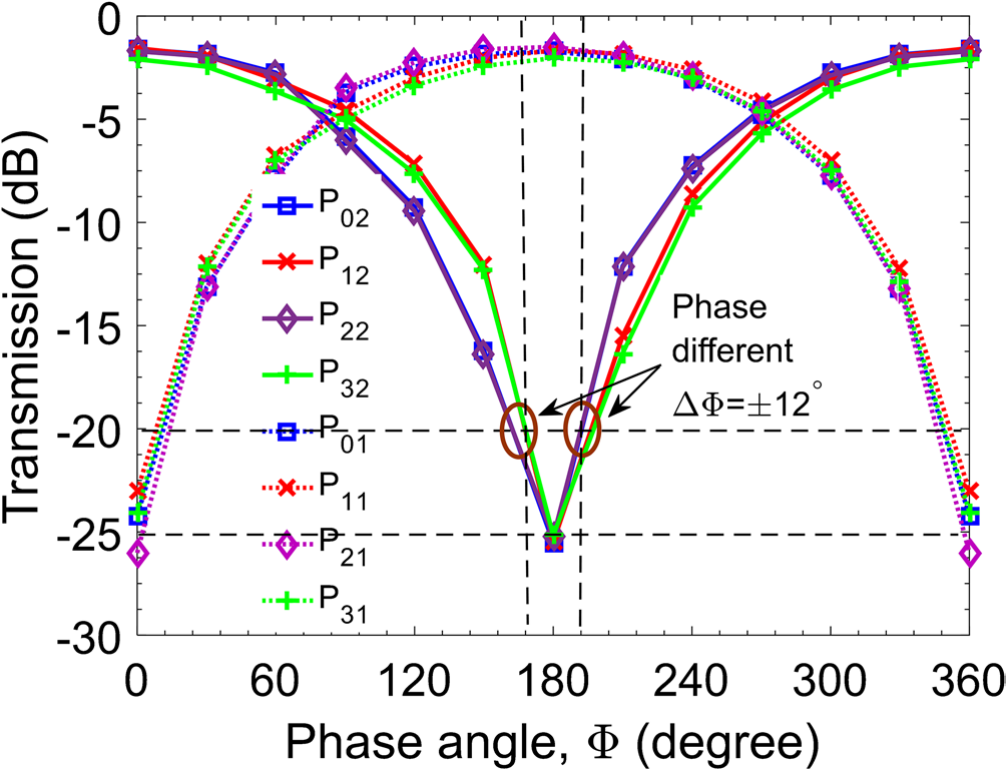
The transmission output powers for four guided modes at different ports as a function of the phase shift angle. Here P_*pn*_ and P_*qn*_ denoted the transmission powers of the mode orders *p* (= 0 or 3) and *q* (= 1 or 2) at the port *n* (= 1 or 2). When the phase is of 0°, 180°, or 360° the outputs are switched. The circles indicate the operating phase range for crosstalked transmission lower than − 20 dB.

Figure 4 shows the output powers $P_{mn} \left( \Phi \right)$ obtained from a numerical simulation using Eq. (7) for four injected modes as a function of phase shift angle, corresponding to bar (straight or ON state) and cross (OFF state) output ports. It is clear to see that the transmission curves are quite the same for all four characteristic modes in the harmonic shape curves. If the phase $\Phi = \pi$ radian, all four modes of TE_0_, TE_1_, TE_2_, TE_3_ will be simultaneously switched to the bar output port, and if $\Phi = 0$ radian, all four modes will be simultaneously switched to the cross output port. In general, at some shifted angle different from an integer number of pi, the optical powers of each guided mode are divided into each output port, on the same side have the same convolution. The power splitting ratio between bar and cross output ports is given by $r_{p} = {\text{tan}}^{2} {(}\frac{{\Phi }}{2}{)}$. In this case, the device plays the role of an arbitrary four-mode power ratio splitting device. In addition, all cases of switching states at the desired output ports are highly efficient with the loss is not exceeded 1.25 dB (the output transmission is larger than 75%) while the transmission at the undesired output ports is lower than − 23 dB.

To control the PSs, we employ the thermo-optic (TO) effect in which the refractive index of the core layer will be modified by thermal excitation resulting in a change of the phase of the propagating lightwave. The phase change can be calculated from the difference of index by^36^:

8 $$\Delta \Phi = k.\Delta n.L_{h} ,$$

where $L_{h}$ is the heater length to obtain the required phase shift of $\Delta \Phi$ and *k* = 2π/*λ* is the wavenumber. The total index change, Δ*n*, is determined by the thermal coefficient, *dn/dT*, of the material, in this study is silicon, and can be represented by linear relation as follows^37,38^:

9 $$\Delta n = K_{c} \cdot \Delta T \cdot \frac{dn}{{dT}},$$

where *K*_*c*_ is the specific heat capacity; $\Delta T = T - T_{0}$ is the temperature change in the silicon waveguide core and *T*_0_ = 300 K; $\frac{dn}{{dT}}$ is the thermo-optic coefficient for silicon material ($\frac{dn}{{dT}} = 1.84 \times 10^{ - 4} {\text{K}}^{ - 1}$ at the 1550-nm wavelength).

In a TO switch, the minimum length to reach the phase shift of $\pm \pi$ radian, which is essential for switching operation, depends on the thermal crosstalk in the gap between the silicon core layer and heaters. A large heat transfer area or large heater length is necessary to make a large shift phase with a small temperature range. However, large geospatial factors will need a large power consumption, thus reducing performance and increasing operating costs. It also causes a large thermal impedance resulting in a high switching time. In addition, a large spatial size also prevents the device from integrating at subwavelength optics as well as on-chip integration.

**Figure 5:**
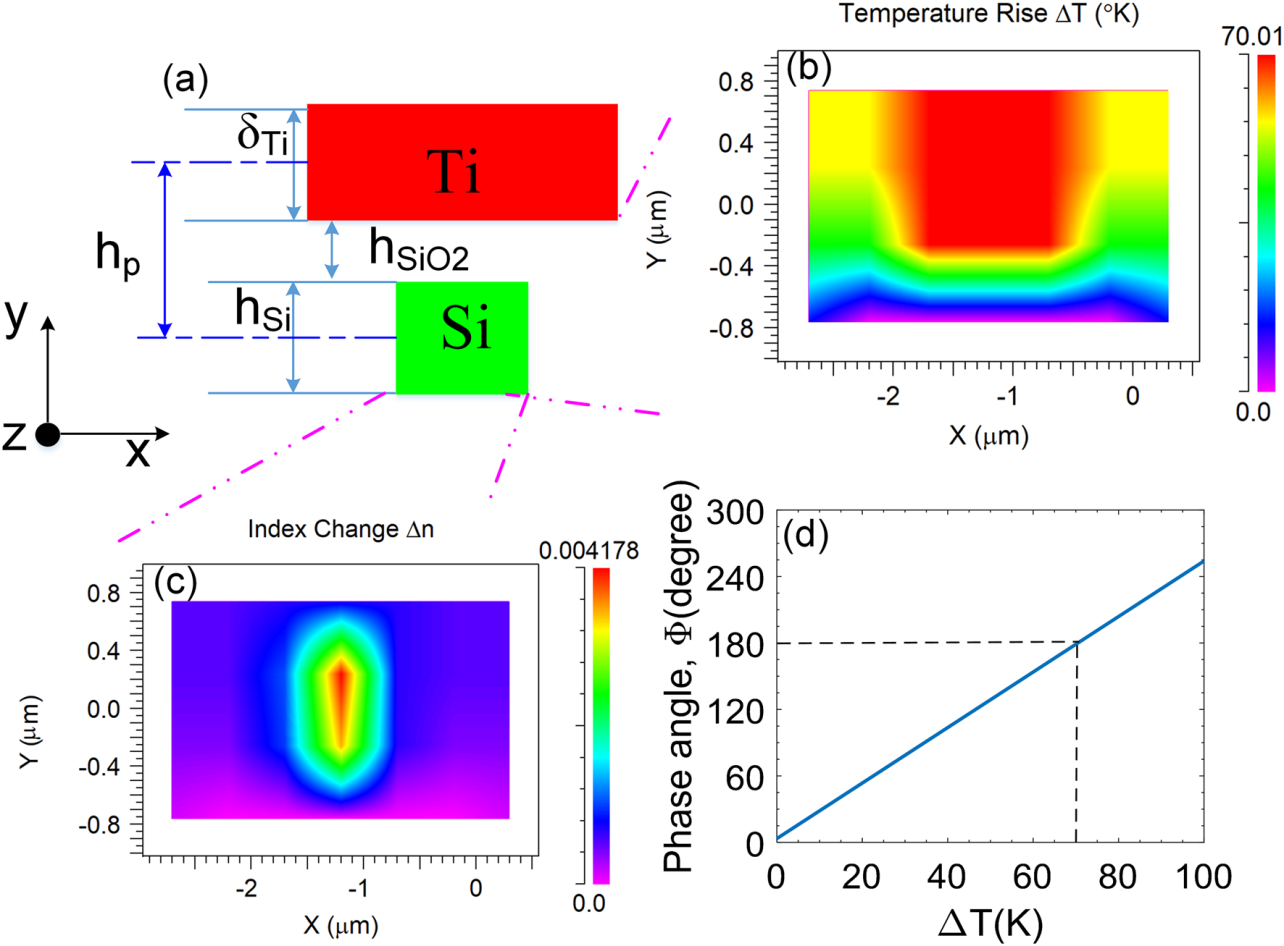
Structure of thermo-optic phase shifter. (**a**) The cross-view with the gap is SiO_2._ Here *δ*_*Ti*_ = 100 nm, *h*_*Si*_ = 220 nm, *W*_*Ti*_ = 1 µm and *h*_*p*_ = 700 nm. (**b**) The x–y plane shows distribution of temperature in the Si core when shifting phase is π radian. (**c**) Index change profile under the thermo-optic effect. (**d**) The simulated phase angle of the TO phase shifter as a function of temperature-change, ΔT, from room temperature.

To reduce the switching time for ultrafast applications, one can use a p–n junction structure as a controllable phase shifter by implanting p++ and n+ + ions of group III/V in silicon ridge waveguide structures^39^. However, such carrier-depletion waveguides suffer a big loss from optical absorption by the ions. To overcome that drawback, in this study, we use driven heaters which are built by coating a metallic thin film of titanium (Ti) and electrical routing pads by coating a layer of aluminum (Al). The designed heaters have a reasonable length of about 200 µm. The driven heaters are placed at the position above the silicon core at a gap of *h*_*p*_ = 0.7 µm. The Ti film layers have a thickness of $\delta_{{{\text{Ti}}}}$ = 100 nm and a width of *W*_*Ti*_ = 1 µm^40^. Figure 5 shows the structure and working principles of the numerically simulated thermo-optic phase shifters. In which, the distance *h*_*p*_ is determined by $h_{p} = h_{Si} /2 + h_{{SiO_{2} }} + \delta_{Ti} /2$, where $h_{{SiO_{2} }}$ is the gap of the upper silica cladding layer sandwiched between the Ti-heater and the silicon core layer, as shown in Fig. 5a. Figure 5b,c shows the distributions of the temperature rise (Δ*T*) and the index change (Δ*n*) in the silicon core layer at the switching state “ON” by using multi-physics tools when the electric power is supplied to reach a required phase difference of π radian. As can be seen the heat distribution mainly focuses on the active region from the micro-heater to the silicon core at access arms and the maximal change is ~ 70 K on the Ti heater surface. The distribution of the index change results in a nonuniform treatment that the highest variation is near the central region below the micro-heater. The phase shift angle increases linearly with the change of the temperature and it equals π radian when the maximum temperature difference increases a quantity of Δ*T* ~ 70 K, as seen in Fig. 5d.

The proposed four-mode switch could be patterned on a silicon-on-insulator (SOI) standard wafer with 220-nm-thick silicon on 3-µm-thick BOX layer of silicon dioxide (SiO_2_) using electron-beam lithography (EBL) and an inductively coupled plasma-induced reactive ion etching (ICP-RIE). A 1-μm-thick SiO_2_ cladding layer will be deposited using a plasma-enhanced chemical vapor deposition (PECVD) process. After this SiO_2_ layer, 100-nm-thick Ti heaters and 1-μm-thick Al contact pads will be deposited by the lift-off process^27^. Then, a 1.5 µm silica layer is deposited using the PECVD process to protect and isolate the silicon device layer. On top of this oxide layer, 100 nm thin film of Ti metal is deposited as a high-resistance heater and 300 nm thin film of aluminum is deposited for the electrical routing using electron-beam evaporation. After the metallization process, a 300 nm SiO_2_ layer is deposited as a protective layer for the heater that is etched away over the aluminum pad for an electrical probing signal. An electrical pulse may apply to drive the controllable phase shifters. This pulse generates heat and is transferred to silicon waveguide changing the refractive index of the silicon core making a phase shift. Currently, TO switches can work with an ultrafast switching time that is shorter than 10 µs^36,41^.

## Characteristics evaluation

**Figure 6:**
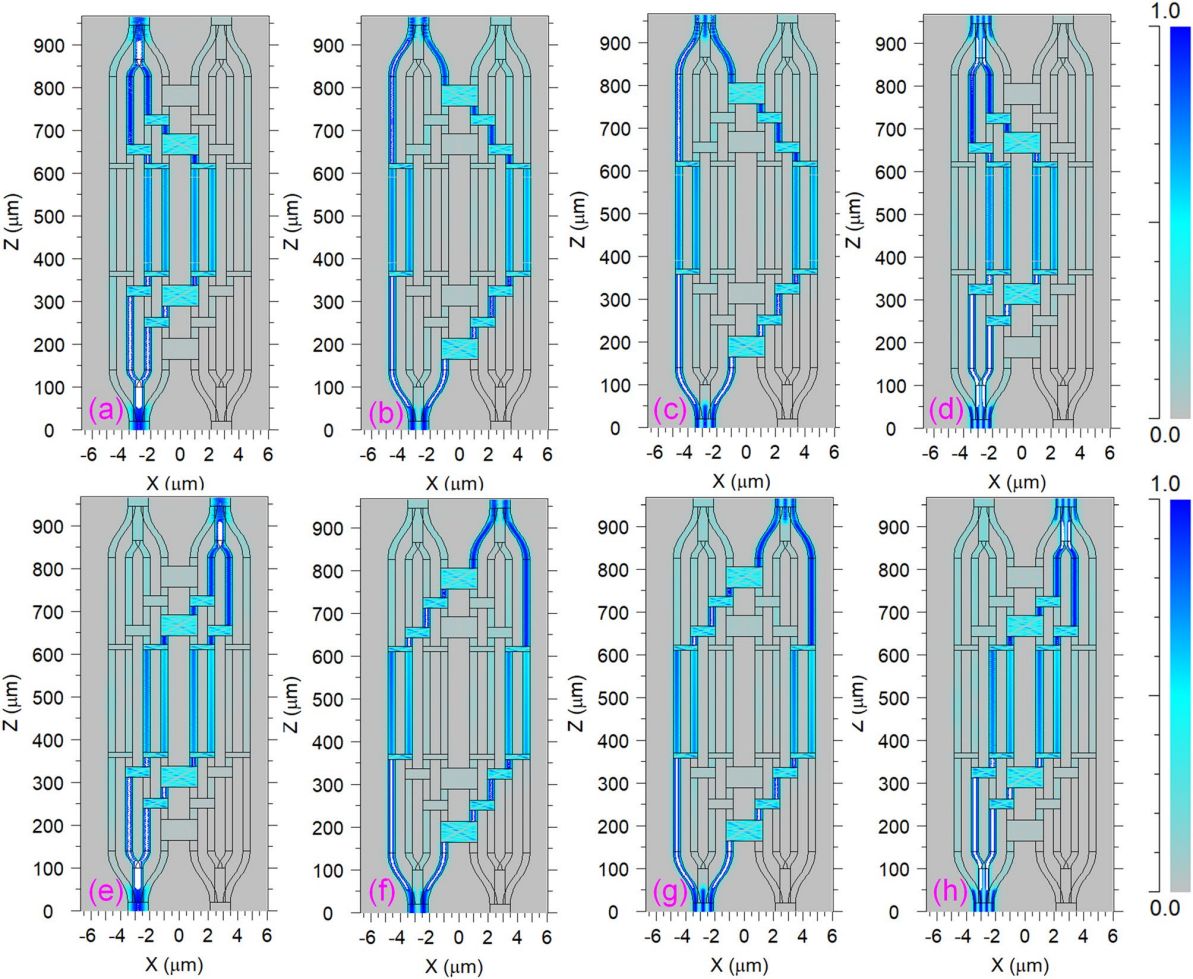
Contour plots of the electric field patterns for the working proposed 2 × 2 four-mode switch: (**a**), (**b**), (**c**) and (**d**) are for TE_0_, TE_1_, TE_2_, TE_3_ modes for ON state, respectively; (**e**), (**f**), (**g**) and (**h**) are for TE_0_, TE_1_, TE_2_, TE_3_ modes for OFF state, respectively.

Figure 6 shows visual images of the electric field distribution that were carried out by numerical simulation for all switching states of four guided modes at the center wavelength of 1550 nm in the bar and cross directions. Simulation results agree with the working principle of the proposed simultaneous 2 × 2 multimode switch as theoretically analyzed. The proposed switch operates at two states: each mode in four input modes entering port I_1_ switched to equivalent order mode on the BAR side at port O_1_ corresponding to “ON” state as seen in Fig. 6a–d. These modes switched to the CROSS side corresponding to the “OFF” state as seen in Fig. 6e–h.

To evaluate the optical performances of the proposed four-mode switch, the key parameters such as insertion loss (I.L) and crosstalk (Cr.T) are studied as a function of operation wavelength that can be calculated by following formulas:

10 $$I.L_{mn} = 10{\text{log}}_{{{10}}} \left( {\frac{{P_{out} }}{{P_{in} }}} \right),$$

11 $$Cr.T_{mn} = 10{\text{log}}_{10} \left( {\frac{{P^{\prime}_{out} }}{{P_{in} }}} \right),$$

where $P_{in}$ is the input power; $P_{out}$ is the output power at the desired output port; $P^{\prime}_{out}$ is the output power at the undesired output port; $n = \left\{ {1,2} \right\}$ stands for the order of output ports O_1_ and O_2_; $m = \left\{ {0,1,2,3} \right\}$ stands for the order of the guided modes when propagating through the device.

**Figure 7:**
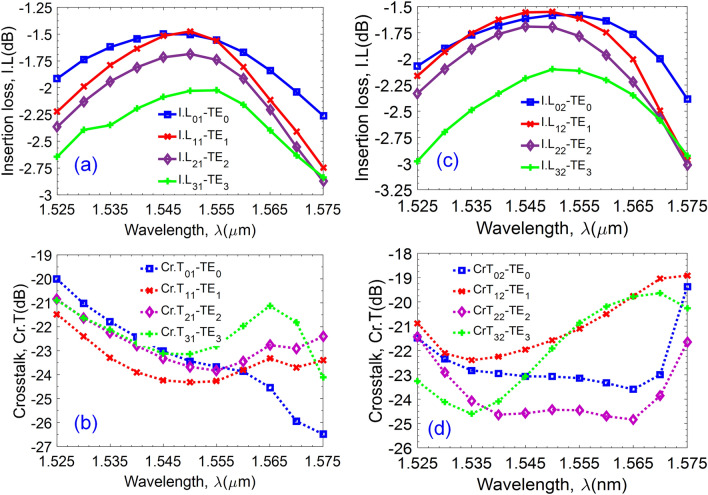
The wavelength-dependent characteristics curves of the optical performance of the device. (**a**) Insertion loss (I.L) and (**b**) crosstalk (Cr.T) for four modes according to the ON switch mechanism from the input I_1_ to the output O_1_. (**c**) Insertion loss and (**d**) crosstalk according to the OFF switch mechanism from the input I_1_ to the output O_2_.

Because of the chromatic dispersion, the mode coupling and mode confinement efficiencies of the optical field are dependent on the operation wavelength. We consider wavelength responses of the insertion loss and crosstalk in the window of 1.525–1.575 µm for the propagation states from the input I_1_ to the output ports O_1_ (Fig. 7a,b) and O_2_ (Fig. 7c,d). As can be seen from these figures, I.L varies from − 1.5 to − 3 dB and Cr.T varies from − 19 to − 27 dB for both cases of ON and OFF states. This low crosstalk demonstrates the excellent performance of the proposed multimode switch in a relatively wide bandwidth of 50 nm indicating advantages of the device in terms of low loss, low crosstalk, and wideband. The MMI couplers constructed for the crossover couplers and the 3-dB couplers have relatively flat responses as a function of the wavelength-spectral response, which the highest transmission attains at the central wavelength of 1550 nm, as shown in Fig. 3a,b. Besides, the suggested multimode switch utilized Y-multijunctions resulting in different wavelength responses for different guided modes. In addition, the operation of the multimode switch depends on the phase shifters. However, the phase difference in the phase shifters is a function of *dn/dT*, and therefore, the required phase difference (π radians) is temperature-dependent and the wavelength-dependent. For each different guided mode at each different wavelength, the temperature needed to transit the phase angle of π radian is different. The proposed device is optimized for working at a central wavelength of 1550 nm. As a result, accumulative transmissions are vigorously rolled off following the wavelength response around the central wavelength of 1550 nm, thus limiting the wavelength bandwidth, as shown in Fig. 7. Noted that, controllable phase shifters are only optimized for the central wavelength 1550 nm, and the 50-nm bandwidth is suitable for an optical switch.

Besides I.L and Cr.T, the figure-of-merit (FOM) criterion is also an important parameter to evaluate device performances. It is well-known that a single-mode system can achieve a good performance of transmission in a wideband, however, there is a limit in spectral efficiency. In contrast, a multichannel system like the MDM–WDM hybridization system has to sacrifice the optical performances for spectral efficiency because of issues on nonlinear effects, high modal crosstalk, and multimode dispersion. Therefore, a photonic integrated circuit (PIC) applying for the MDM–WDM systems needs to consider the optimized performance in order to get a high FOM value for two aspects of wavelength range and number of guided modes. There are some different definitions of FOM used in designing the PICs, such as FOM standards for the TO switches versus material^42^, consumption power^43^, geometrical size^44^, etc. However, in this design, our FOM standard is applied for optimizing optical performances on the number of guided mode and wavelength, which is defined as follows^45^:

12 $$FOM = 1 - \left( {1 - \alpha } \right).\frac{1}{2M}.\sum\limits_{n = 0}^{3} {\left| {x_{n} - \frac{1}{2}} \right| - \alpha .\frac{1}{2M}.\sum\limits_{n = 0}^{3} {\left| {y_{n} } \right|} } ,$$

where *M* is the wavelength resolution scanned in the range from 1.525 to 1.575 µm. *α* is the factor with the value is within the range ($0 \le \alpha \le 1$) as a trade-off between I.L and Cr.T; *x, y* are corresponding to I.L and Cr.T, respectively.

**Figure 8:**
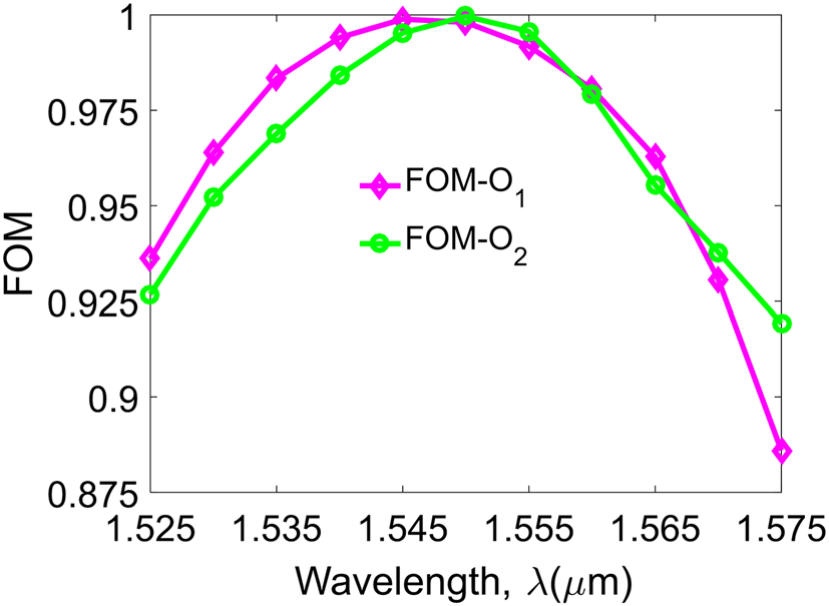
Figure-of-merit. Simulated FOM as a function of the operating wavelength for two states: ON, output O_1_ (purple) and OFF, output O_2_ (green) in the wavelength window of 50 nm.

Equation (12) satisfies the following inequality: 0 < FOM ≤ 1. It will be ideal if FOM reaches unity. Figure 8 shows that FOM is ideal at the operation wavelength of 1550 nm. In the wavelength range of 1525–1575 nm, FOM can get the value from 0.92 to 1 for the switching state I_1_O_1_ (ON state) and from 0.88 to 1 for the switching state I_1_O_2_ (OFF state). This demonstrates the high performance of the device in the wideband of 50 nm of the third telecom windows, especially at the central wavelength of 1550 nm.

**Figure 9:**
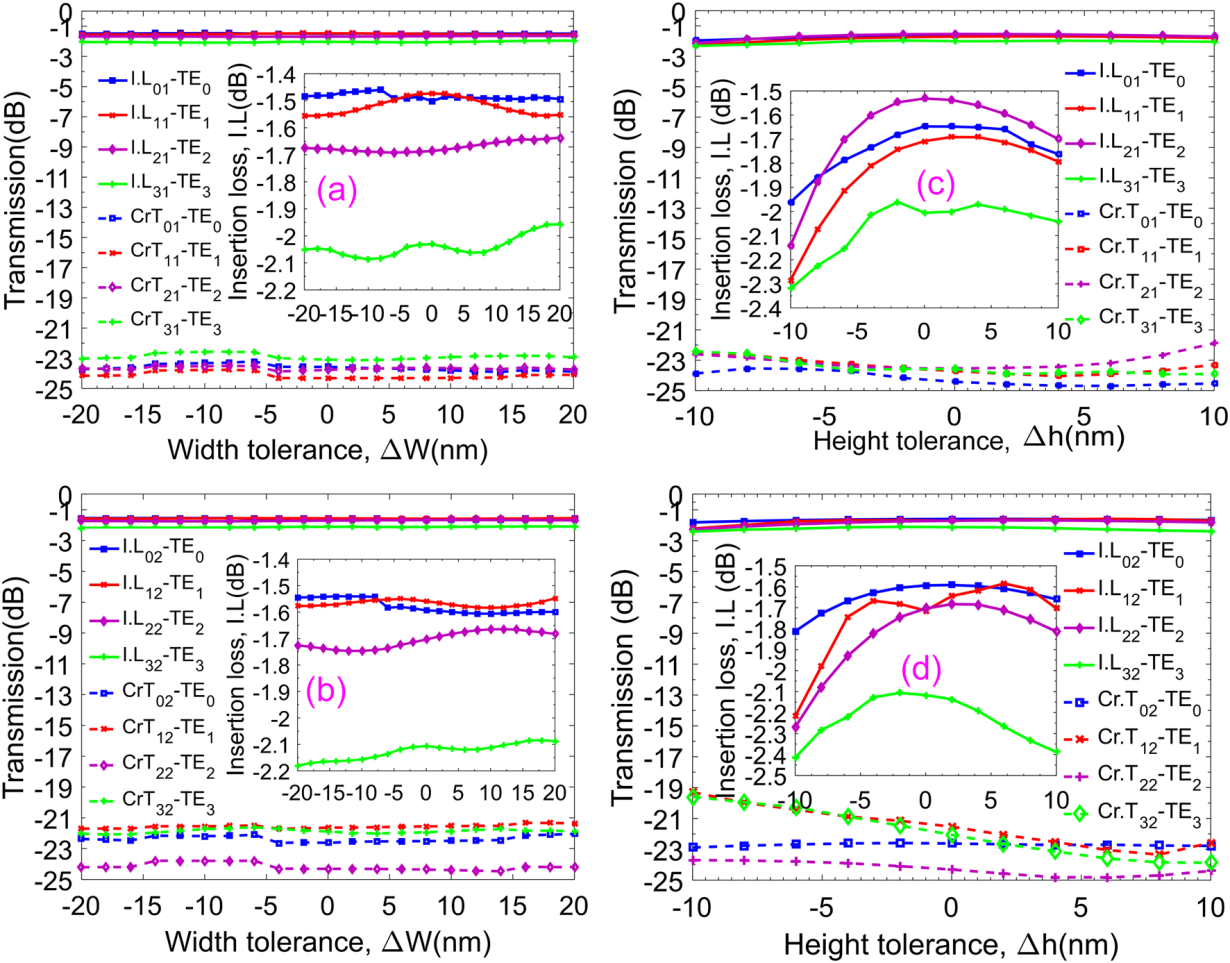
Insertion loss and crosstalk of the proposed device for four operation modes. For the ON state (**a**), from the input I_1_ to the output O_1_ and OFF state (**b**), from the input I_1_ to the output O_2_, as a function of a width tolerance *ΔW*. For the ON (**c**) and OFF (**d**) states as a function of height tolerance *Δh*.

Fabrication tolerances are also important for designing photonic devices, especially for simulation-based designs. Because errors of material and geometrical parameters strongly affect the working performances. For example, the quality of SOI wafer depends on suppliers in terms of geospatial tolerances, high wall roughness, the purity of silicon, crystallinities, etc. Besides, the accuracy of fabricated patterns strongly depends on electron–beam writing or deep ultraviolet (DUV) photolithography technologies. Also, the simulation tolerances are depending on the accuracy of the simulation models as well as the simulation algorithms. Therefore, we investigated the tolerances for the proposed switch in terms of the waveguide width and waveguide height. The width tolerances *ΔW* are shown in Fig. 9a,b for two outputs corresponding to the ON and OFF states when the guided modes are injected into the input I_1_ with a change of the width within ± 20 nm. Simulated data shows that I.L fluctuates very little around − 1.4 dB to − 1.7 dB while Cr.T keeps stable with the value is smaller than − 21 dB for four guided modes. Also, the high tolerances, *Δh,* presented in Fig. 9c,d show I.L fluctuates around − 2 dB and Cr.T is less than − 19 dB for both switching states ON and OFF. These tolerances are investigated in the variation of the height tolerances equally as ± 10 nm. Such relatively high tolerances on the aspects of geometrical dimensions are acceptable thanks to the current advancement of fabrication technology in the-state-of-the-art electron–beam writing.

The power consumption of the TO phase shifters is often evaluated by the essential power to achieve a phase shift of π radians ($P_{\pi }$). This is a crucial measure parameter in the operation of a thermo-optic optical switch. It is desirable to obtain the product of $P_{\pi } .\tau = H.\Delta T_{\pi }$ as an optimal value during the operation process of the optical switch^36^. Here, *H* stands for the heat capacity, Δ*T*_*π*_ is the temperature change from a cold state to a hot state to attain the expected phase shift, and $\tau$ is the switching time related to the fall time or the rise time in the phase shifter temporal response. The switching electric consumption power is determined by the following equation utilizing a modified two-dimensional treatment of the heat flow on the lateral spreading^46^:

13 $$P_{\pi } = \frac{{\lambda \kappa_{{SiO_{2} }} \left( {\frac{{W_{PS} }}{{h_{{SiO_{2} }} }} + 0.88} \right)}}{{\left| {\frac{dn}{{dT}}} \right|_{Si} }},$$

where $\kappa_{{SiO_{2} }}$ = 1.4 W/(m K) is the thermal conductivity of SiO_2_, *λ* is the operation wavelength, and *W*_*PS*_ is the width of the Ti-metal film on the lateral direction. The switching time can be considered as a consequence of the required transport time of the heat flow propagation from the micro-heater to the silicon core layer along the active length of the TO phase shifter relating to the consumption power by the relation^47,48^:

14 $$\tau = \frac{{\pi \lambda \rho_{{SiO_{2} }} C_{{SiO_{2} }} A}}{{eP_{\pi } \left| {\frac{dn}{{dT}}} \right|_{Si} }}$$

where $\rho_{{SiO_{2} }}$ = 2.203 g/cm^3^ is density of silica,$C_{{SiO_{2} }}$ = 0.703 J/(g K) is specific heat capacity of silica;$A = (2L_{th} + W_{PS} )(h_{Si} + h_{{SiO_{2} }} )$ is the effectively heated cross-section area relating to geometry parameters of the TO phase shifter; *h*_*Si*_ = 220 nm is the height of the silicon core; *L*_*th*_ is the thermal diffusion length measured by taking the distance where the maximum temperature laterally decreased at 1/*e*^2^ away from the silicon waveguide.

From Exps (13), (14) the switching time can be deduced as:

15 $$\tau = \frac{{\pi \rho_{{SiO_{2} }} C_{{SiO_{2} }} (2L_{th} + W_{PS} )(h_{Si} + h_{{SiO_{2} }} )}}{{e\kappa_{{SiO_{2} }} \left( {\frac{{W_{PS} }}{{h_{{SiO_{2} }} }} + 0.88} \right)}}$$

**Figure 10:**
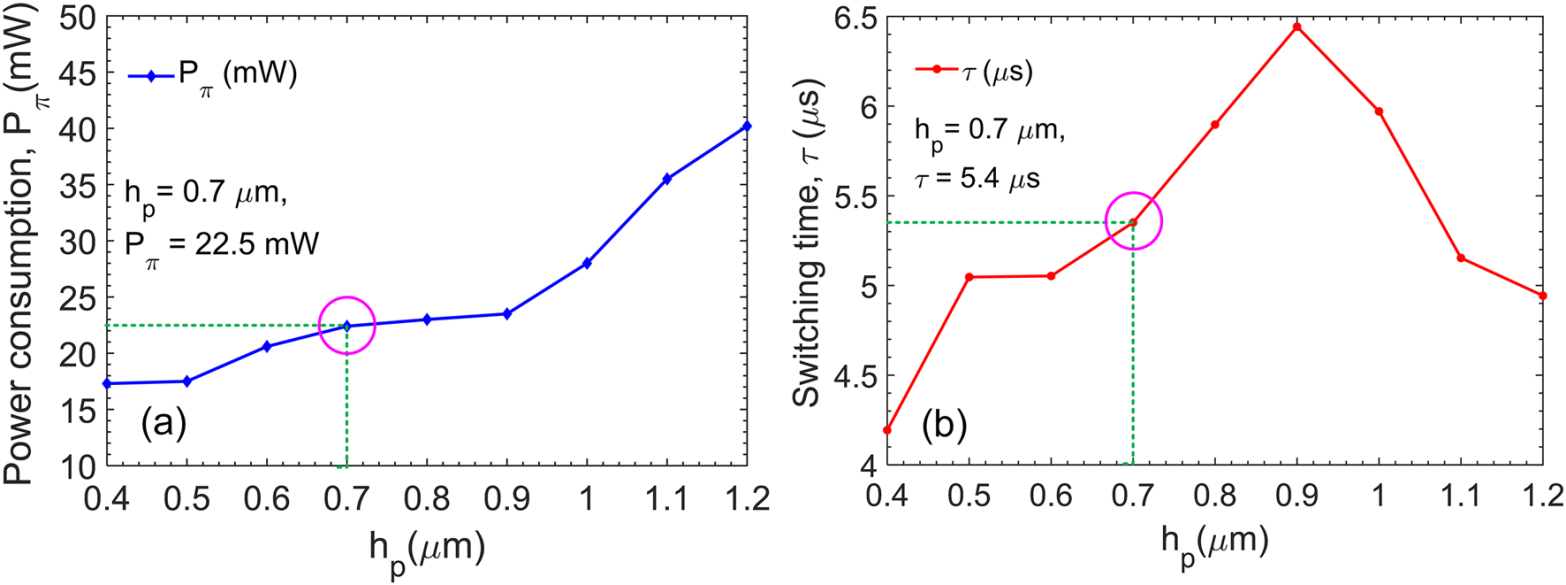
Electric power consumption and switching time. The simulated power consumption (**a**) at the switching level and the switch time (**b**) of the phase shifter as a function of the distance *h*_*p*_ between the Ti heater and the silicon core layer. The circles indicate the values at *h*_*p*_ = 0.7 µm corresponding to $h_{{SiO_{2} }}$ = 540 nm.

Besides, the trade-off between power consumption and switching speed is unavoidable as well as integrated size and long range propagation. For the TO optical switch, switching time is designed for several microseconds, and therefore the electric power consumption should be kept in a few tens of mW^40^. Figure 10 exhibits the simulated electric power consumption and the switching time as a function of the distance *h*_*p*_. As can be seen, the electric power needed to achieve a phase shift of π radians increase when the distance *h*_*p*_ increases. However, the switching time increases to the maximum value of 6.4 µs at *h*_*p*_ to 0.9 µm and decreases when *h*_*p*_ increases further. At the selected physical distance of *h*_*p*_ = 0.7 µm (or $h_{{SiO_{2} }}$ = 0.54 µm), *P*_*π*_ obtained a relatively small value of 22.5 mW with a switching time of 5.4 µs.

**Figure 11:**
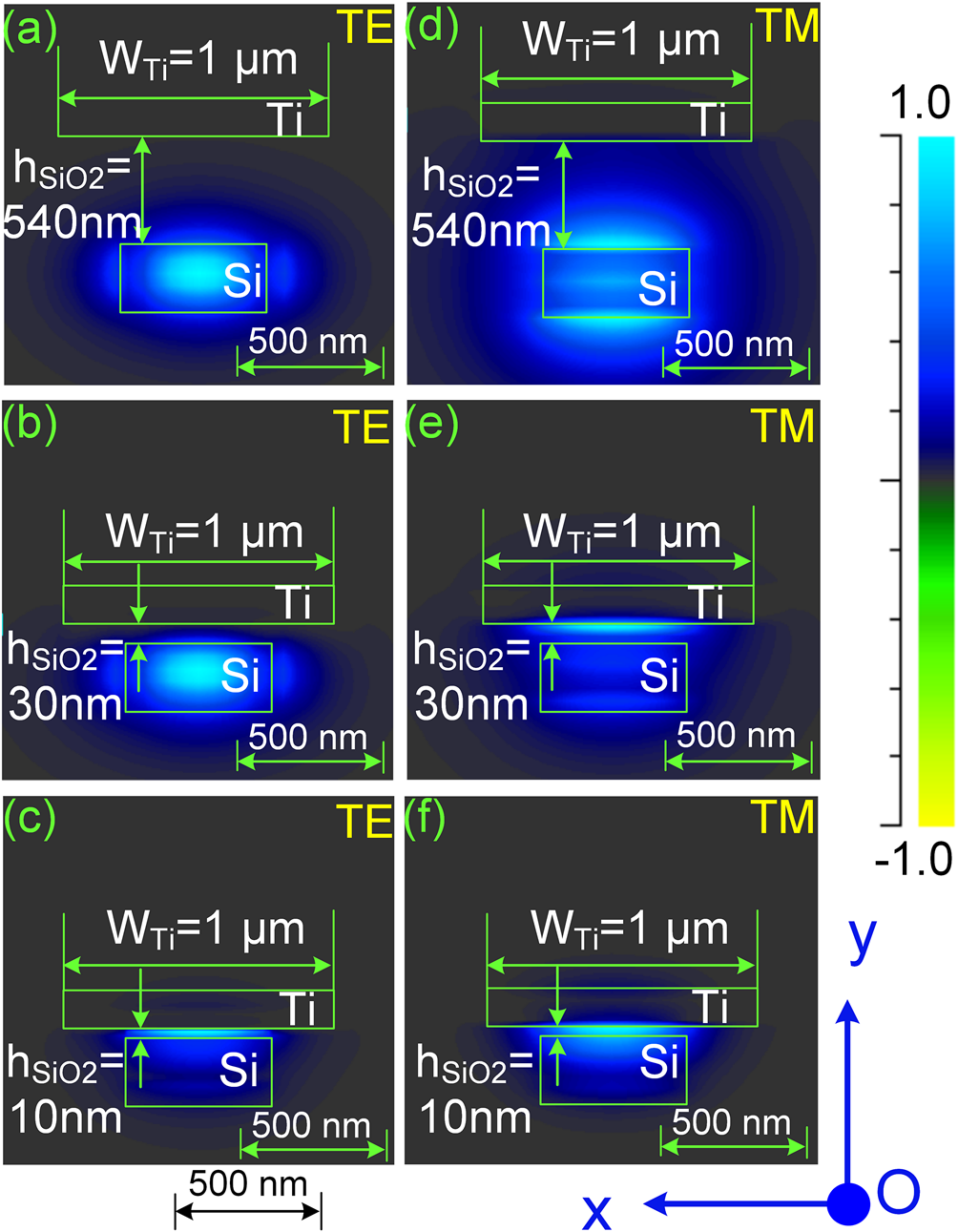
Mode profile (|Ey|) in Ti heater-based phase shifter. Modes simulated by the FEM method for both TE and TM polarization states at some gaps, $h_{{SiO_{2} }}$, of 540 nm, 30 nm, and 10 nm at *λ* = 1550 nm.

To reduce the power consumption *P*_*π*_ as well as the desired temperature difference (Δ*T*_*π*_) in the switching operation, the length of the thermo-optic phase shifters must be increased, thus making the PS length larger and also switching time slower. Furthermore, the power consumption can be reduced when the gap between the metallic heater and the silicon core decreases but should be properly chosen minimizing the impact of the plasmonic effect on the propagation of lightwave in term of phase shift and optical loss. In the plasmonic regime, the *p*-polarized optical modes (TM modes) is confined along the metal–dielectric interface. Hence, the Ti-heater strongly affects the TM modes than the TE modes in term of hybrid plasmonic effect when the gap between the Ti-heater and the silicon core is smaller than a limit. This is depicted in Fig. 11 where mode profiles of both TE and TM polarization states are simulated by the finite element method (FEM) for several gaps of $h_{{SiO_{2} }}$ corresponding to 540 nm, 30 nm, and 10 nm at the wavelength *λ* = 1550 nm. Simulation results show that the plasmonic mode is significant when $h_{{SiO_{2} }} \le$ 30 nm for TM modes and only becomes significant when $h_{{SiO_{2} }} \le$ 10 nm for TE modes. When optical fields are in the plasmonic modes, the imaginary part of the dielectric constant becomes larger leading to a larger conductive absorption. This effect restricts the propagation length of the optical fields in the photonic device. In our proposed optical switch, which was designed for the operation of TE modes with the distance *h*_*p*_ = 700 nm ($h_{{SiO_{2} }}$ = 540 nm), the impact of plasmonic effect can be neglected because the optical fields are always preserved in photonic modes.

## Discussion

The proposed device is a 2×2 multimode switch enabling the simultaneously four-mode switching operation in a multimode waveguide for the MDM and hybrid WDM–MDM applications. In order to couple the switch with the WDM–MDM system, grating couplers^49,50^ or edge-couplers^51,52^ are required to couple high-order modes from the few-mode fibers to the silicon waveguides for realizing the functionality of a wavelength division multiplexed few-mode fiber switch. In another way, subwavelength gratings (SWG) consisting of periodically arranged dielectric particles with dimensions much smaller than the wavelength are utilized for highly efficient coupling from single-mode fibers to a silicon single-mode waveguide-based chip in a compact footprint over a broad wavelength bandwidth^53,54^. Then, fundamental modes in single-mode silicon waveguides are multiplexed into a multimode waveguide and vice versa by mean of MMI couplers^41,55^, ring resonators^3,56^, or adiabatic couplers^57^ before connecting to the multimode switch. Finally, single-mode optical fibers are coupled to the WDM system via the WDM multiplexer following the ITU-T G.694.1 recommendation to complete a hybrid WDM–MDM system. In another application scenario, single-mode optical fibers carrying each dedicated wavelength are coupled to each dedicated silicon single-mode waveguide via curved Bragg gratings allowing the propagation of light in the third telecom windows of 1550-nm at first. Then, a set of individual wavelengths are multiplexed into a single-mode silicon waveguide carrying the total traffic of a WDM-channels group via an arrayed waveguide grating (AWG)^58^. Next, each group of the single-mode silicon waveguide is multiplexed to a multimode bus waveguide employing the phase-matched technique as adiabatic couplers^57^. Finally, four groups into a four-mode waveguide are connected to an input/output port of the designed 2×2 four-mode switch. The constructed structure can permit the switching operation of four WDM groups in the 2×2 configuration. Recently, adiabatic couplers are popularly used for multiplexing low-order modes into a multimode waveguide that such structures can carry up to dozens of optical modes in a multimode waveguide^9,59,60^.

Compared to some related recent studies on multimode switches, our proposed switching device either supports the operation of four guided modes instead of two guided modes^27^ or has much simpler and more compact than those reported elsewhere^61,62^. To the best of our knowledge, this novel design and demonstration by numerical simulation in the proof-of-concept of the 2 × 2 four-mode switch has not been proposed before. The device enables the switching operation for simultaneous four guided modes of transverse electric polarization states without the need of high order modes exchange and complicated spatial switching structures. Moreover, the proposed optical switch can operate as a multifunctional-multimode processing device, such as an arbitrary ratio multimode power splitter that can be realized with only one control bit for diving the multimode power simultaneously. In addition, the proposed structure potentially be used as a building block to scale up the higher number of guided modes.

## Conclusions

In conclusion, we have designed and optimized a novel 2 × 2 four-mode optical switch using numerical simulations for the first time. The switch can operate in a 50 nm wide wavelength band from 1525 to 1575 nm with the low insertion loss and crosstalk that are lower than − 3 and − 19 dB, respectively. We also pointed out that the hybrid plasmonic effect between the metallic heater and the Si waveguide of the phase shifter does not effect to the device performance in the chosen parameters. In addition, the device has relatively large geometry tolerances correspondingly to ± 20 nm and ± 10 nm of width and height tolerances, respectively. Furthermore, the device is very compact in a small area of 10 μm × 960 μm offering a huge potential for applications in very large scale integrated photonic circuits as well as in mode and wavelength division multiplexing switching systems.
